# Correction: Functional double dissociation within the entorhinal cortex for visual scene-dependent choice behavior

**DOI:** 10.7554/eLife.28407

**Published:** 2017-05-11

**Authors:** Seung-Woo Yoo, Inah Lee

Yoo S-W, Lee I. 2017. Functional double dissociation within the entorhinal cortex for visual scene-dependent choice behavior. *eLife* **6**:e21543. doi: 10.7554/eLife.21543.

Published 07, February 2017

Two of the visual stimuli (peacock feather and palm tree patterns) in Figure 2A were used in the SNSC task, but not in the SSC task. We have corrected this error by replacing those two images with correct ones (bamboo and mountain patterns). Please note that this correction does not affect the results and conclusions of the original paper.

The Corrected Figure 2 is shown here:

**Figure 1:**
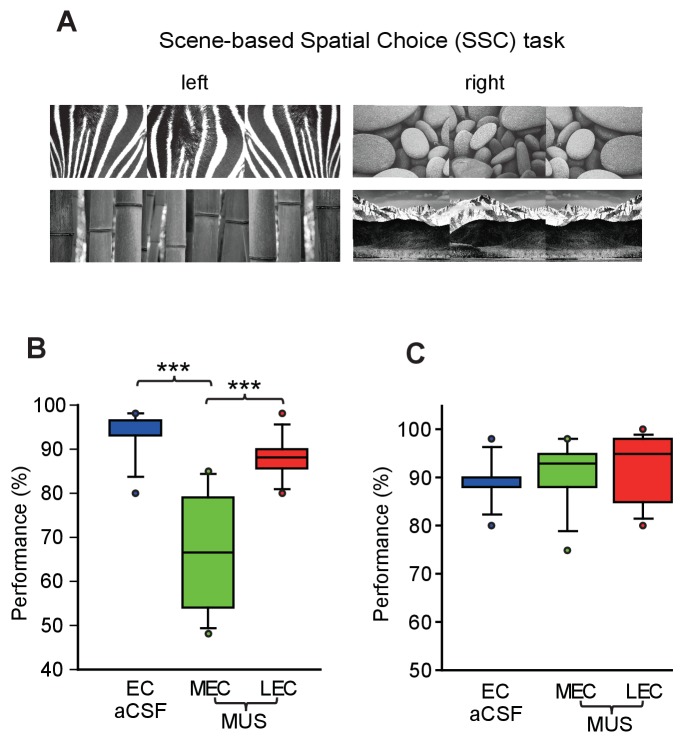

The originally published Figure 2 is also shown for reference:

**Figure 2:**
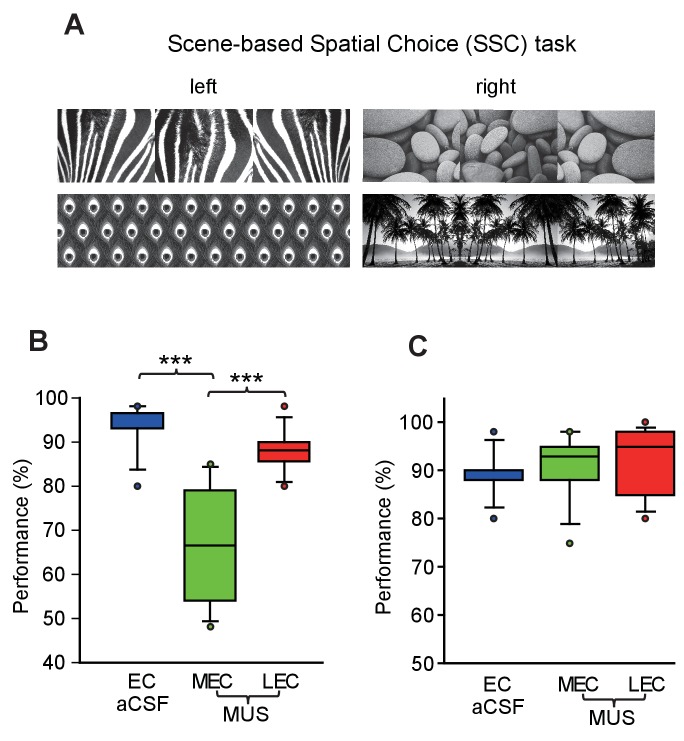

The article has been corrected accordingly.

